# Curative Power of Hot Springs

**Published:** 1876-03

**Authors:** 


### CURATIVE POWER OF HOT SPRINGS

Fourteen years ago we had charge of a Government hospital in South America. Many of the patients were suffering from chronic diseases of various kinds, and some of them had been in the hospital for years, a burden and expense to the nation.

We suggested to the American Minister that it would be an act of humanity toward the sufferers and result in great saving to the Government, if provision were made to send certain patients to the Hot Baths of the "Cauquenies" for treatment, as the French and English surgeons were doing with certain patients in the hospitals. The minister heartily concurred with us, and requested us to visit the baths and carefully investigate the plan of treatment and its results.

The baths are situated in the "Heart of the Andes," far up toward the region of perpetual snow. There was an excellent hotel capable of accommodating 200 guests. With the exception of three or four adobé huts near the hotel there was no other human habitation within eighteen miles. The hotel was crowded with invalids and pleasure-seekers.

We commenced our investigations promptly by examining the baths and by leading all the invalids we met into conversation concerning their ailments and the results of the treatment. These reports were decidedly favorable in almost every case. Among the guests at the hotel was a gentleman whom we shall remember with feelings of gratitude while we live. In answer to our usual question "Why are you here?" he quietly replied, "From gratitude." Seeing that the reply did not satisfy us, he explained that for more than ten years he had been a sufferer from an internal, inflammatory disease, which, in its chronic form, was supposed to be incurable. That four years previously he came to the baths and was treated for thirty days and entirely cured. That from that time there had not been the slightest return of the disease, and he was in the enjoyment of perfect health; but that he intended to visit the baths every year as long as he lived, from feelings of "gratitude" for his recovery. Had it been in his power to bestow upon us the wealth of Golconda, instead of this rehearsal of his experience, it would not have rejoiced us half so much. We, too, had been for years a sufferer from the same malady, and had sought the best medical advise in Europe and America, but none gave promise of relief. All treatment had been simply palliative, and all hope of a cure had long since been abandoned. To be raised in a moment from hopeless invalidity to the near prospect of full and permanent health, is a sensation that language is inadequate to describe.

We tooks our baths and our "sweats" faithfully for about thirty days. During that time all the symptoms were apparently worse than ever, and we left the baths feeling that our last hope had "turned to ashes." We "threaded" our way along that lonely path beside the mountain stream that leads out of the wilderness, feeling that if we ever returned it would not be from "gratitude."

It may have been a month after our return from the baths when we one day discovered that we were well. We had suffered so long and so constantly, and this condition had become so natural to us, and we had so little hope of ever being better, that we did not take note of the exact time when recovery was complete; but certain it is that the baths cured us, and that the cure was radical and complete; for since the day we first noticed that we were well again there has not been the least return of the old malady, and we have not had an hour's illness of any kind.

We believe that the baths of the Cauquenies will radically and permanently cure a majority of all cases of rheumatism of whatever type, diseases of the liver and spleen, dyspepsia, chronic diarrhœa, chronic inflammation of the bladder and other internal organs, scrofulous affection, skin diseases, nervous and gouty affections, and many others; but the Hot Springs of the Cauquenies are more than five thousand miles from New York, which makes them inaccessible to nearly all invalids residing here.

We send many patients to the Hot Springs of Arkansas every year. Evidently these baths are not inferior to the Cauquenies, and the reports of their curative power are highly satifactory. But these springs are more than one thousand miles from New York. The arrangement of the baths is rude and the hotel accommodations are inferior. From favorable reports reeeived from various sources we have been led to investigate the Hot Springs of Virginia,and the result of this investigation is highly satisfactory. We have found that the analysis of the water is almost identical with that of the hot springs of the Cauquenies, and that the temperature of the warmest spring is the same—that is, 110° Fahr. We find, also, that the same diseases are treated with the same satisfactory results. The waters of the Hot Springs of Arkansas are of a higher temperature than those of Virginia, but before they can be used for baths they must be cooled down to 110° Fahr. to be endurable, so that in respect to temperature they have no advantage over the Virginia baths, but rather the disadvantage of parting with some of their essential qualities in the process of cooling. There are other medicinal springs in the vicinity of the Hot Springs of Virginia that are in great favor with the public; but for the treatment of the diseases before referred to we prefer the hot springs. The hotel accommodations and bath houses are unsurpassed by any in the country. The physician in charge is Prof. J. L. Cabell of the University of Virginia. He is one of the most eminent physicians in this country. It seems, then, that everything has been done to make the Hot Springs of Virginia one of the most desirable resorts for health or pleasure-seekers to be found in this country. We have such confidence in the power of natural hot baths to overcome so many painful and—if not properly treated—fatal diseases, that we feel it our duty to make their advantages known as far as possible.

For nearly all chronic diseases there is no other treatment that will produce such wonderful results. In most cases a proper medical treatment of a few days, before taking the baths, will render their favorable action more certain. We have seen cases where the bath seemed to have no effect when this preliminary treatment had been neglected. For example: persons who have been injured by the unwise use of mercury may depend on having the mercury eliminated from the system by the baths, provided they have undergone a treatment by iodide of po. tassium before taking the baths. The baths alone will be found insufficient to effect a cure. In all cases, therefore, it is best to seek the advice of a competent physician.

